# *In Vitro* Neuroprotective Activities of Compounds from *Angelica shikokiana* Makino

**DOI:** 10.3390/molecules20034813

**Published:** 2015-03-16

**Authors:** Amira Mira, Shuntaro Yamashita, Yoshinori Katakura, Kuniyoshi Shimizu

**Affiliations:** 1 Division of Systematic Forest and Forest Products Sciences, Department of agroenvironmental sciences, Faculty of Agriculture, Graduate School of Kyushu University, Fukouka 812-8581, Japan; E-Mail: dramiramera@yahoo.com; 2 Department of Pharmacognosy, Faculty of Pharmacy, Mansoura University, Mansoura 35516, Egypt; 3 Department of Bioscience & Biotechnology, Graduate School of Bioresource and Bioenvironmental sciences, Kyushu University, Fukouka 812-8581, Japan; E-Mails: shuntaro@grt.kyushu-u.ac.jp (S.Y.); katakura@grt.kyushu-u.ac.jp (Y.K.)

**Keywords:** *Angelica shikokiana*, neuroprotection, amyloid β peptide, hydrogen peroxide, acetylcholine esterase

## Abstract

*Angelica shikokiana* is widely marketed in Japan as a dietary food supplement. With a focus on neurodegenerative conditions such as Alzheimer’s disease, the aerial part was extracted and through bio-guided fractionation, fifteen compounds [α-glutinol, β-amyrin, kaempferol, luteolin, quercetin, kaempferol-3-*O*-glucoside, kaempferol-3-*O*-rutinoside, methyl chlorogenate, chlorogenic acid, hyuganin E, 5-(hydroxymethyl)-2-furaldehyde, β-sitosterol-3-O-glucoside, adenosine (isolated for the first time from *A. shikokiana*), isoepoxypteryxin and isopteryxin] were isolated. Isolated compounds were evaluated for *in vitro* neuroprotection using acetylcholine esterase inhibitory, protection against hydrogen peroxide and amyloid β peptide (Aβ_25-35_)-induced neurotoxicity in neuro-2A cells, scavenging of hydroxyl radicals and intracellular reactive oxygen species and thioflavin T assays. Quercetin showed the strongest AChE inhibition (IC_50_ value = 35.5 µM) through binding to His-440 and Tyr-70 residues at the catalytic and anionic sites of acetylcholine esterase, respectively. Chlorogenic acid, its methyl ester, quercetin and luteolin could significantly protect neuro-2A cells against H_2_O_2_-induced neurotoxicity and scavenge hydroxyl radical and intracellular reactive oxygen species. Kaempferol-3-*O*-rutinoiside, hyuganin E and isoepoxypteryxin significantly decreased Aβ_25-35_-induced neurotoxicity and Th-T fluorescence. To the best of our knowledge, this is the first report about neuroprotection of hyuganin E and isoepoxypteryxin against Aβ_25-35_-induced neurotoxicity.

## 1. Introduction

Alzheimer’s disease (AD) is a neurodegenerative and age-related disease that gradually worsens over time and accounts for 60%–80% of causes of dementia. It is characterized by progressive cognitive impairment, memory loss and behavioral changes [1]. The pathology of the disease is associated with decreased cholinergic response, increased oxidative stress and aggregation of Aβ (amyloid β) peptides. AD is associated with selective death of cholinergic neurons, specifically in areas of the brain that mediate learning, memory and cognition functions, resulting in deficiencies in the neurotransmitter, acetylcholine (ACh). Inhibition of acetylcholinesterase (AChE), the enzyme responsible for the hydrolysis of ACh, elevates the levels of ACh and consequently, improves the disease severity [2]. On the other hand, so-called senile plaques composed of Aβ-peptides are the hallmark of AD [3]. The deposition of soluble Aβ leads to the aggregation of peptide-forming protofibrils and oligomers that induce neural cell death [4,5]. Although, the initial source of oxidative stress in AD is still unclear, multiple studies have associated oxidative stress and free radical damage to the etiology of AD. It is reported that brains with AD have high levels of H_2_O_2_, hydroxyl and oxygen radicals that peroxidize membrane lipids and oxidize proteins producing drastic cellular damage [6,7]. Limited treatment remedies emphasize the importance of developing effective strategies for diminishing AD burden. As there is a continuous upsurge in searching for the treatment from medicinal plants, *Angelica shikokiana* (*A. shikokiana*) was investigated for the presence of neuroprotective compounds that could inhibit AChE enzyme or decrease the neurotoxicity of oxidative damage induced by H_2_O_2_ or Aβ-peptides in mouse neuroblastoma Neuro-2A cells.

*A. shikokiana* (Apiaceae) is a perennial herb known as Inutoki in Japan, where it is widely marketed as a dietary food supplement in the form of a health tea preparation which is used as a substitute for ginseng roots [8] for treating digestive and circulatory-system diseases [9] hyperlipidemia and aging symptoms [10]. Our previous study [11] showed that ethanol extract of the aerial part (stems and leaves) of the plant had neuroprotection against H_2_O_2_-induced neurotoxicity in neuro-2A cells and acetylcholine esterase inhibition. Other *Angelica* species such as *A. sinensis* [12] and *A. dahuricae* [13] were previously studied for neuroprotection, but to the best of our knowledge, there is no previous report about any neuroprotective effects of *A. shikokiana*, or its bioactive compounds, so the present work aimed to characterize the neuroprotective compounds of the aerial parts to provide scientific evidence for supporting the use of *A. shikokiana* as a herbal tea for improving memory and other age related diseases.

## 2. Results and Discussion

**Figure 1:**
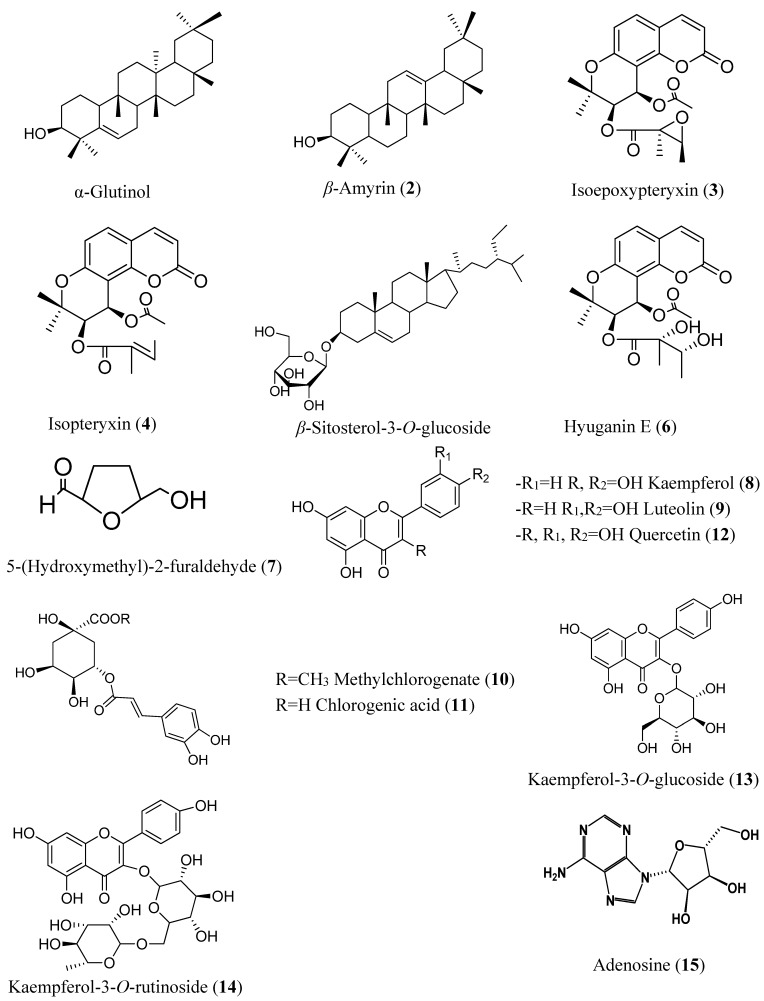
Structures of compounds isolated from the aerial parts of *A. shikokiana*.

Bio-guided fractionation and isolation (Flowchart 1) of the methanol extract of the aerial part of *A. shikokiana* resulted in the isolation of fifteen compounds (Figure 1). Their structures were elucidated by co-chromatography with authentic samples and comparison of their NMR spectra with previously reported datas; two triterpenes: α-glutinol (**1**) [14] and β-amyrin (**2**) [15]; three coumarins: isoepoxypteryxin (**3**), isopteryxin (**4**) [8] and hyuganin E (**6**) [16]; five flavonoids: kaempferol (**8**), luteolin (**9**), quercetin (**12**) [17], kaempferol-3-*O*-glucoside (**13**) [18] and kaempferol-3-*O*-rutinoside (**14**) [19]; two phenolic acids: methyl chlorogenate (**10**) [20] and chlorogenic acid (**11**) [21] and others; β-sitosterol-3-*O*-glucoside (**5**) [15], 5-(hydroxymethyl)-2-furaldehyde (**7**) [22] and adenosine (**15**) [23]. All compounds except isoepoxypteryxin and isopteryxin were isolated for the first time from *A. shikokiana.* All compounds were evaluated for neuroprotection using AChEI and protection against H_2_O_2_ and Aβ_25-35_-induced neurotoxicity in neuro-2A cells.

**Flowchart 1:**
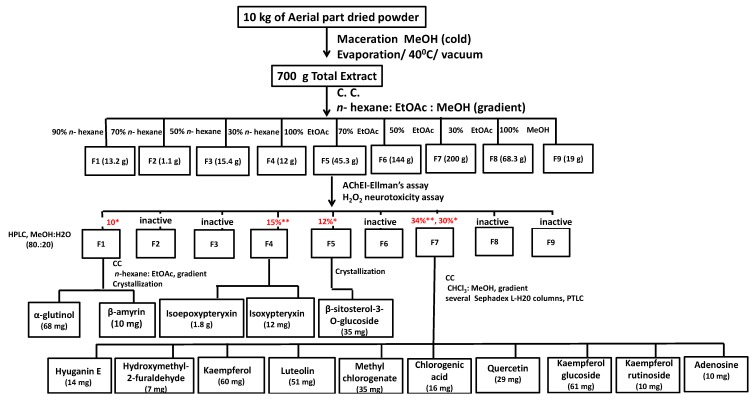
Scheme of bio-guided fractionation and isolation of neuroprotective compounds of methanol extract of the aerial part of A. shikokiana. * % neuroprotection against H_2_O_2_-induced neurotoxicity in neuro-2A cells. ** % AChEI at 400 µg/mL.

### 2.1. Acetylcholine Esterase Inhibitory Assay

Inhibition of AChE was evaluated using Ellman’s method (results are shown in Table 1). Flavonoid and coumarin compounds had the strongest inhibitory activity. Quercetin (**12**) showed the highest inhibition followed by flavonoid glycosides kaempferol-3-*O*-rutinoside (**14**) and kaempferol-3-*O*-glucoside (**13**) (IC_50_: 35.5, 50.4 and 80.4 µM, respectively) while kaempferol (**8**) and luteolin (**9**) showed lower activities. The coumarin compound, huganin E (6) showed moderate activity (IC_50_: 286.5 µM) while isoepoxypteryxin (**3**) and isopteryxin (**4**) showed lower activities (IC_50_: 327.4 and 475.9 µM, respectively).

**Table 1:** IC_50_ values of the isolated compounds in the AChEI assay ^1^.

Compound	IC_50_ (µM)	Compound	IC_50_ (µM)
α-Glutinol (**1**)	inactive	Luteolin (**9**)	more than 500 (30% at 250 µM) ^3^
β-Amyrin (**2**)	inactive	Methyl chlorogenate (**10**)	more than 500 (10% at 500 µM)
Isoepoxypteryxin (**3**)	327.4 ± 3.4	Chlorogenic acid (**11**)	inactive
Isopteryxin (**4**)	475.9 ± 1.5	Quercetin (**12**)	35.5 ± 1.3
β-Sitosterol-3-*O*-glucoside (**5**)	inactive	Kaempferol-3-*O*-glucoside (**13**)	80.4 ± 5.1
Hyuganin E (**6**)	286.5 ± 2.1	Kaempferol-3-*O*-rutinoside (**14**)	50.4 ± 0.4
5-(Hydroxymethyl)-2-furaldehyde (7)	inactive	Adenosine (**15**)	inactive
Kaempferol (**8**)	more than 500 (30% at 250 µM) ^2^		

^1^ Galantamine (positive control) IC_50_ = 1.4 ± 0.7 µM. ^2,3^ At higher concentrations; Non-linear relationship between higher concentration and enzyme activity was observed.

**Figure 2:**
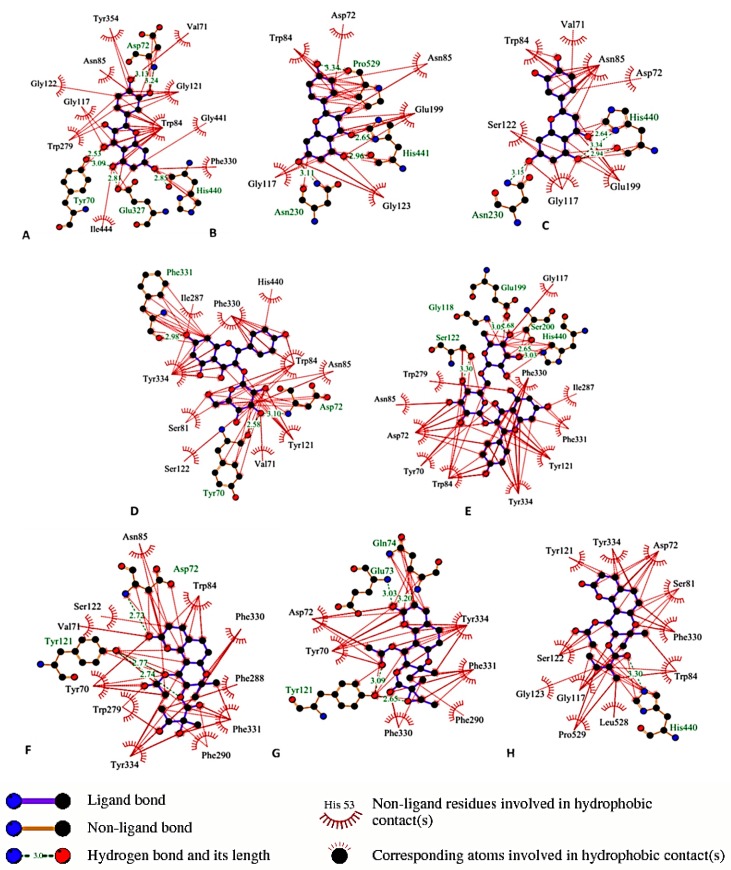
Binding residues of the active compounds in AChE assay using LigPlot+ v.1.4 program. (**A**) quercetin (**12**); (**B**) kaempferol (**8**); (**C**) luteolin (**9**); (**D**) kaempferol-3-*O*-glucoside (**5**) (**E**) kaempferol-3-*O*-rutinoside (**14**); (**F**) isoepoxypteryxin (**3**); (**G**) isopteryxin (**4**); (**H**) hyuganin E (**6**).

To gain insights about the binding interaction of the active compounds with the active site of AChE, molecular docking experiments were performed for compounds that showed activity in the *in vitro* assay (docking scores are shown in Table 2). It is well established that the hydrolysis reaction of ACh by AChE is mediated by two sites: the anionic site that withdraws ACh molecules to the second site; the esteratic site; at which hydrolysis occurs [24]. X-ray crystallography of Torpedo and mammal AChE showed that the esteratic site consists of five residues containing the catalytic triad (Ser-200, Glu-327, His-440), Phe-288 and Phe-290. The esteratic site lies at the bottom of a narrow gorge (20 Å), which consists of 14 aromatic residues, among them Trp-84 and Phe-330, which contain anionic sites. Asp-72, Tyr-121, Tyr-70, Tyr-354, and Trp-279 are the residues of another anionic site, the peripheral anionic site (PAS) [25,26]. Binding of inhibitors to the residues of PAS causes a change of AChE conformation and prevents ACh from passing through the narrow gorge [27]. Another important component of the active center in AChE is the oxyanion hole which is formed by peptidic NH groups from Gly118, Gly119, and Ala201 and has an important role in stabilization of high-energy intermediates and the transition state through hydrogen bonding [28]. As shown in Figure 2, quercetin (A) (**12**) could bind through H-bonds to the His-440 (at catalytic site) and Tyr-70 (at anionic site) residues. Its more potent activity compared to kaempferol (B) (**8**) and luteolin (C) (**9**) could be explained by additional H-bonds between the C-5 OH group and Glu-327 (at the catalytic site) and C-3'OH group and Asp-72 (at the anionic site). H-bonds between the rhamnose moiety of the sugar part of kaempferol-3-*O*-rutinoside (E) (**14**) and His-440, Ser-200 (at the catalytic site), Gly-118 (oxyanion hole) and H-bonds between glucose moiety of kaempferol glucoside (D) (**13**) and Asp-72 and Tyr-70 (at the anionic site) may account for the superior activity of the two glycosides over kaempferol (**8**). Coumarins showed binding affinity to anionic site residues. For example, isopteryxin (G) (**4**) could bind through hydrophobic bonds to Asp-72, Tyr-70, and Phe-330 and through H-bond to Tyr-121. Isoepoxypteryxin (F) (**3**) showed similar binding in addition to one more hydrophobic bond to Trp-84 residue. The higher activity of hyuganin E (H) (**6**) could be explained through its ability to bind to one of the catalytic triads; His-440 (H-bond) as well as to anionic site residues; Asp-72, Tyr-121, Trp-84 and Phe-330.

**Table 2:** Docking score of active compounds in AChEI assay.

Compound	Binding Energy (kcal/mol)	Compound	Binding Energy (kcal/mol)
Quercetin (**12**)	−129.1 ± 0.7	Kaempferol-3-*O*-rutinoside (**14**)	−195.6 ± 0.3
Kaempferol (**8**)	−119.3 ± 0.3	Isoepoxypteryxin (**3**)	−128.6 ± 0.3
Luteolin (**9**)	−122.1 ± 0.7	Isopteryxin (**4**)	−129.9.2 ± 0.8
Kaempferol-3-*O*-glucoside (**13**)	−166.4 ± 0.4	Hyuganin E (**6**)	−132.2 ± 0.7

**Figure 3:**
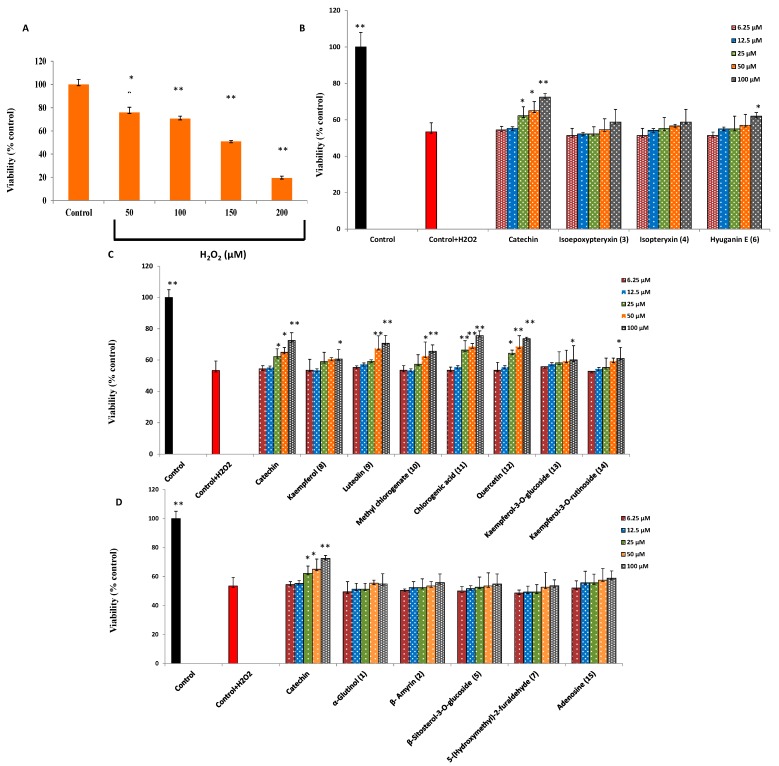
(**A**) Dose-dependent cytotoxic effects of H_2_O_2_ on Neuro-2A cells. (**B**–**D**) neuroprotection against H_2_O_2_-induced neurotoxicity in neuro-2A cells by isolated coumarins, phenolic compounds and others, respectively. Values are represented as means ± standard deviations (SD), *n* = 5. * and ** Significant difference from control in (A) and from cell viability of H_2_O_2_ (150 µM) treatment in (B, C, D) at *p* < 0.05 and *p* < 0.01, respectively.

### 2.2. Protection against H_2_O_2_-Induced Neurotoxicity and Scavenging of Hydroxyl Radicals and Intracellular ROS

The neurotoxicity of H_2_O_2_ was determined by a MTT reduction assay after 24 h cell incubation with different concentrations of H_2_O_2_. As shown in Figure 3A, H_2_O_2_ at 50 to 200 µM showed a dose-dependent decrease in cell viability. 150 µM of H_2_O_2_, caused 48.2% ± 1.8% cytotoxicity so, it was used to determine the cytoprotective effect of isolated compounds. The maximum concentration of the compounds that cause no significant cytotoxicity to the cells after 2 h incubation was found to be 100 µM (data not shown). Thus, neuro-2A cells were treated with different concentrations (6.25, 12.5, 25, 50, 100 µM) of isolated compounds. Chlorogenic acid (**11**), quercetin (**12**), luteolin (**9**), and methyl chlorogenate (**10**) showed dose-dependent neuroprotection, and, at 100 μM, resulted in significant increase in cell viability of 22% ± 2.6%, 20% ± 3.9%, 17% ± 6.7% and 12% ± 2.6%, respectively (Figure 3B). Hyuganin E (**6**), Kaempferol (**8**), Kaempferol-3-*O*-glucoside (**13**) and kaempferol-3-*O*-rutinoside (**14**) showed only significant (*p* < 0.05) increase in cell viability at 100 µM. Other compounds did not show any neuroprotective activity.

**Figure 4:**
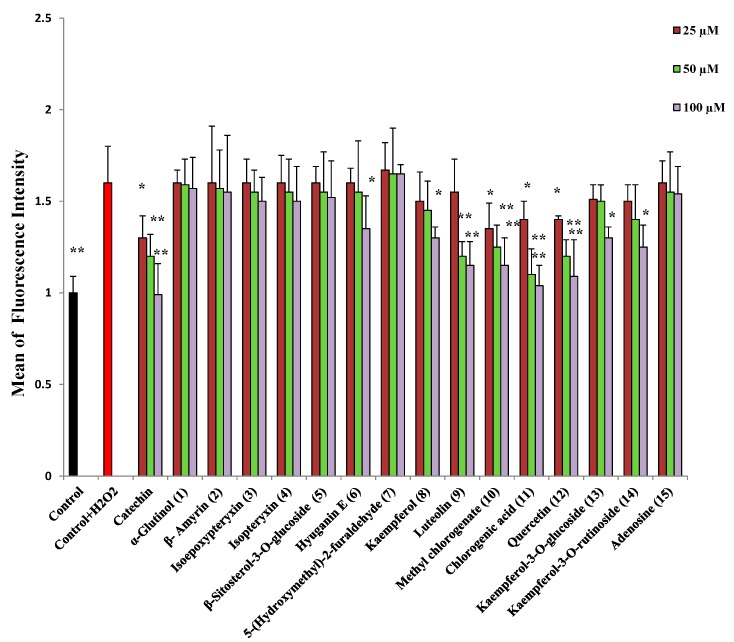
Fluorescence intensity of neuro-2A cells treated with different concentrations of sample and treated with H_2_O_2_ (600 µM) for 30 min. All values are means ± SDs (*n* = 3). * and ** Significant difference from fluorescence intensity of H_2_O_2_ treatment at *p* < 0.05 and *p* < 0.01, respectively.

Isolated compounds were investigated for their ability to scavenge hydroxyl radicals and intracellular reactive oxygen species. Compounds with a catechol moiety namely, chlorogenic acid (**11**), quercetin (**12**), luteolin (**9**), and methyl chlorogenate (**10**), at concentrations of 50 and 100 µM, could significantly decrease the fluorescence intensity of DFF (Figure 4), indicating their ability to scavenge intracellular ROS and decrease oxidative stress. Other compounds, namely hyuganin E (**6**), kaempferol (**8**) and its glycosides could decrease the fluorescence intensity at the higher concentration of 100 µM. Similarly, catechol moiety containing compounds were able to scavenge hydroxyl radicals (Table 3) and protect 2-deoxyribose from degradation. Coumarins and triterpenes showed very weak or no activity at 300 μM, their maximum solubility). It was observed that IC_50_ values for the non-site-specific assay was lower than IC_50_ values for the site-specific one, indicating that the activity may be related to hydroxyl-scavenging activity more than metal-chelating activities. 

**Table 3:** Activity of isolated compounds for hydroxyl radical scavenging in the 2-deoxyribose degradation assay.

Sample	IC_50_ (μM)	
	Non Site-Specific	Site-Specific
Catechin (positive control)	473 ± 8.6	534 ± 19.2
Kaempferol (**8**)	720 ± 7.9	901 ± 17.9
Luteolin (**9**)	753 ± 11.1	980 ± 15.8
Methyl chlorogenate (**10**)	840 ± 12.4	920 ± 10.9
Chlorogenic acid (**11**)	602 ± 8.9	809 ± 13.7
Quercetin (**12**)	672 ± 10.6	837 ± 21.6
Kaempferol-3-*O*-glucoside (**13**)	1150 ± 6.7	1304 ± 24.3
Kaempferol-3-*O*-rutinoside (**14**)	1030 ± 25.7	1540 ± 20.8
Adenosine (**15**)	-	-
**Sample**	at 300 µM * (% Inhibition)	
	**Non Site-Specific**	**Site-Specific**
α-Glutinol (**1**)	-	-
β-Amyrin (**2**)	-	-
Isoepoxypteryxin (**3**)	6 ± 1.4%	-
Isopteryxin (**4**)	10 ± 2.1%	-
β-Sitosterol-3-*O*-glucoside (**5**)	-	-
Hyuganin E (**6**)	37.5 ± 3.5%	-
5-(Hydroxy methyl)-2-furaldehyde (**7**)	4 ± 0.3%	-

* Maximum solubility in phosphate buffer.

### 2.3. Protection against Aβ_25-35_ Induced Neurotoxicity and Thioflavin T Assays

**Figure 5:**
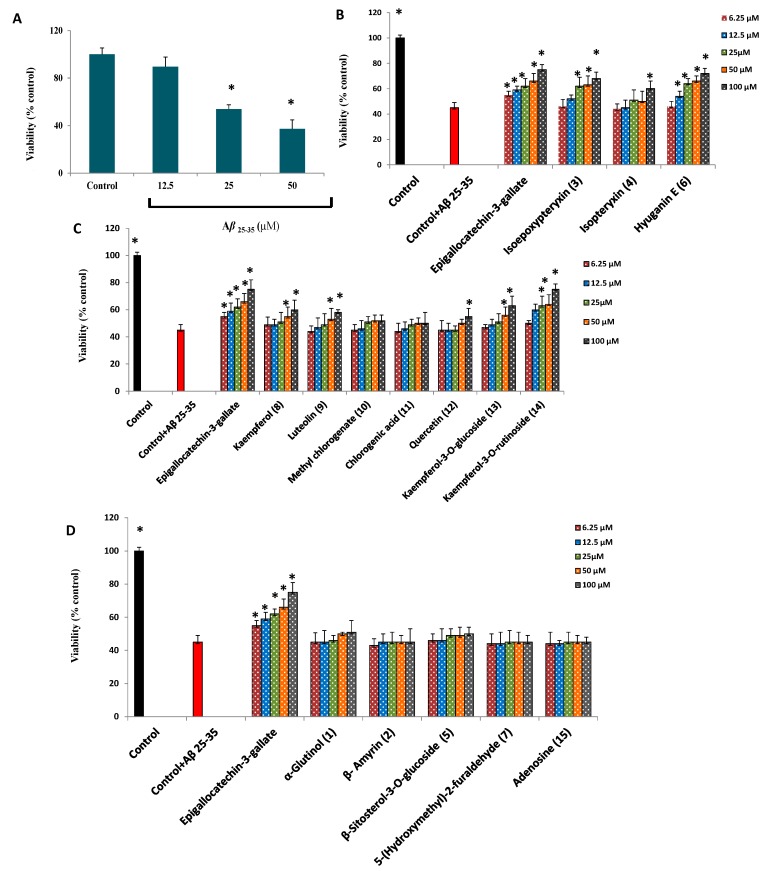
(**A**) dose-dependent cytotoxic effects of Aβ_25-35_ on Neuro-2A cells. (**B**–**D**) neuroprotection against Aβ_25-35_ (25 µM) induced neurotoxicity in neuro-2A cells by isolated coumarins, phenolic compounds and others, respectively. All values are means ± SDs (*n* = 5). * Significant difference from control in (A) and from cell viability of of Aβ_25-35_ (25 µM) treatment at *p* < 0.05.

Kaempferol-3-*O*-rutinoside (**14**) and the coumarin compounds; hyuganin E (**6**) and isoepoxypteryxin (**3**) showed dose-dependent neuroprotection against Aβ_25-35_-induced neurotoxicity (Figure 5B,C) and increased cell viability by 30% ± 6.8%, 27% ± 5.3%, and 23% ± 2.5%, respectively, at 100 µM compared with cells treated with Aβ_25-35_ alone, which caused 55% ± 4.6% cytotoxicity after 72 h incubation. Isopteryxin (**4**), kaempferol (**8**), luteolin (**9**), quercetin (**12**) and kaempferol-3-*O*-glucoside (**13**) showed significant neuroprotection (*p* < 0.05) at the highest concentration (100 µM). 

**Figure 6:**
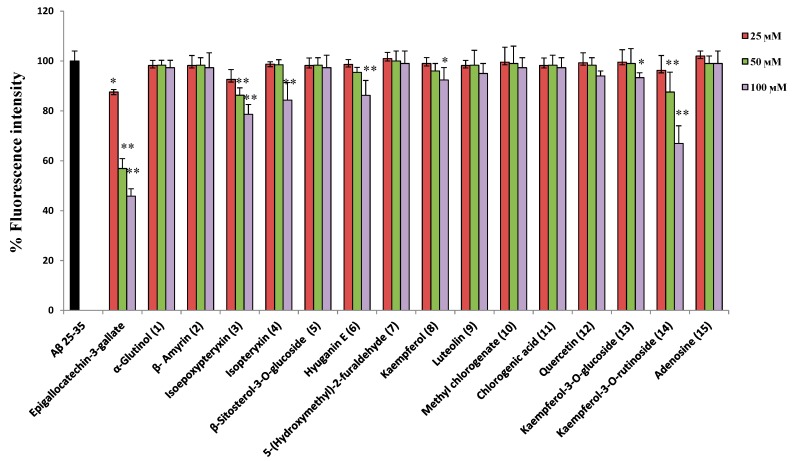
Effect of isolated compounds on Aβ_25-35_ aggregation determined by measuring thioflavin T fluorescence. All values are means ± SDs (*n* = 4). * and ** Significant difference from fluorescence intensity of Aβ_25-35_ treatment at *p* < 0.05 and *p* < 0.01, respectively.

In our study, the neuroprotective compounds against Aβ_25-35_ induced neurotoxicity, didn’t show strong activity for scavenging hydroxyl or intracellular ROS radicals, which means that the mechanism of protection is different from that of the anti-oxidant activity of the compounds. Furthermore, there are controversial studies about the pro-oxidant [29] and antioxidant [30] role of amyloid β protein in AD. Hence, we found that the most promising strategy to understand the mechanism of action of active compounds is to investigate the ability of compounds to interact directly with Aβ to prevent fibril aggregate formation [31] using Th-T assay. Aggregated Aβ_25-35_ (25 µM), prepared by incubation at 37 °C for 24 h with shaking in 50 mM PBS, had strong fluorescence with Th-T. So, isolated compounds at different concentrations were incubated under the aggregating conditions with Aβ_25-35_. Figure 6 shows that kaempferol-3-*O*-rutinoside (**14**), hyuganin E (**6**), and isoepoxypteryxin (**3**) decreased Th-T fluorescence in a dose-dependent manner. Kaempferol-3-*O*-rutinoside (**14**) and isoepoxypteryxin (**3**) decreased the fluorescence intensity significantly (33% ± 2.5% and 12% ±3.7% and 21% ± 3.4%, 13% ± 1.8% at concentrations of 100 and 50 µM, respectively). The decrease of fluorescence intensity of Th-T could be due to the ability of the active compounds to inhibit Aβ_25-35_ aggregation [32]. Isopteryxin (**4**), kaempferol (**8**) and kaempferol-3-*O*-glucoside (**13**) showed weak activity at the highest tested concentration. 

The results of kaempferol-3-*O*-rutinoside (**14**) match previous findings [32] related to its ability to protect against amyloid peptides induced neurotoxicity and inhibit their aggregation. As it is well established that binding of Aβ fibrils to PAS of AChE accelerates their aggregation [33,34], the ability of kaempferol-3-*O*-rutinoside (**14**) as well as active coumarins like hyuganin E (**6**), and isoepoxypteryxin (**3**) to inhibit Aβ aggregation may be also due to their ability to bind to PAS as previously outlined in the docking results. The mechanism of protection may be also due to other pathways such as the ability of active compounds to bind to amyloid aggregates masking the surface responsible for their toxicity or to disrupt the already formed aggregates to form aggregates off-pathway with lower cytotoxicity [31] and those mechanisms need further investigation. To the best of our knowledge, this is the first report about neuroprotection of hyuganin E and isoepoxypteryxin against Aβ_25-35_-induced neurotoxicity.

Although Fraction 1 (Flowchart 1) showed 11% neuroprotection against H_2_O_2_-induced neurotoxicity at 400 µg/mL, it was observed that its isolated compounds, α-glutinol (**1**) and β-amyrin (**2**), did not show significant neuroprotection in examined assays. This could be explained by the presence of other minor compounds responsible for the activity and this matter requires further investigation. 

## 3. Experimental Section 

### 3.1. General

The dried powder of the aerial part of *A. shikokiana* (voucher specimen No. Y-1) was obtained from Tateyama Motors Corporation (Kurume, Japan). Mouse neuroblastoma neuro-2A cell lines were purchased from Riken Bioresource Center (Ibaraki, Japan). Eagle’s Minimum Essential Medium (EMEM) was purchased from Nissui Pharmaceutical (Tokyo, Japan). Fetal bovine serum (FBS) was obtained from Gibco BRL (Tokyo, Japan). Thiazolyl blue tetrazolium bromide (MTT), galantamine hydrobromide, Aβ_25-35_, thioflavin T, 2-deoxyribose and AChE from *Electrophorus electricus* (electriceel), 500 U/mg, were purchased from Sigma (St. Louis, MO, USA). Acetylthiocholine iodide (ACTI) was purchased from Tokyo Chemical Industry (Tokyo, Japan). 5,5-Dithiobis[2-nitrobenzoic acid] (DTNB) and Bes-H_2_O_2_-AC was obtained from Wako (Osaka, Japan). All other chemicals were purchased from Merck (Darmstadt, Germany).

### 3.2. Bio-Guided Isolation 

Ten kg of the aerial part of *A. shikokiana* were extracted with methanol (15 L × 4, 25 °C) and the total extract (700 g) was fractionated using silica gel column chromatography (50 × 19 cm i.d., 3.5 kg, *n*-hexane: EtOAc, 100: 0 to 0:100, gradient and EtOAc/MeOH, 100:0 to 0:100, gradient, 1 mL/min) into nine fractions (F1-9). Bio-guided fractionation was conducted using AChEI and protection against H_2_O_2_ induced neurotoxicity in neuro-2A cells assays. Fractions showed inhibition for AChE more than 10% at 400 µg/mL (F4 and F7) and or protection against H_2_O_2_ neurotoxicity more than 10% at 400 µg/mL (F1, F5 and F7) were chosen for isolation of their bioactivities. 

F1 (13 g, eluted 10% EtOAc, 11% neuroprotection) was subjected to silica gel column (35 × 3 cm i.d., 300 kg, *n*-hexane—EtOAc 100:0 to 0:100, gradient, 0.5 mL/min). Similar fractions were collected together on the basis of similarity in R*_f_* values. Active fractions were purified by repeated crystallization from MeOH to afford compounds **1** and **2** (68 mg, 10 mg, respectively). 

F4 (12 g, eluted 70% EtOAc, 15% AChEI) was purified using HPLC (Inertsil ODS-3, 20 × 250 mm, MeOH: H_2_O, 80:20, isocratic, flow rate, 5 mL/min; injection volume, 1 mL, 100 mg/mL, λ = 254, 366 nm). Peaks appearing with retention times of 19 and 30 min were collected separately and concentrated using vacuum drying at 45 °C and completely dried using a freeze dryer to yield compounds **3** and **4** (1.8 g, 12 mg).

F5 (45.3 g, eluted 10% EtOAC, 12% neuroprotection) yielded white crystals which were further purified by crystallization to afford compound **5** (35 mg). 

F7 (200 g, eluted with 50% MeOH, 34% AChEI and 30% neuroprotection) was subjected to silica gel column chromatography (47 × 3 cm i.d., 300 g, CHCl_3_: MeOH, 100:0 to 0:100, gradient, 0.5 mL/min). Similar fractions were pooled together on the basis of similarity in R*_f_* values. Those fractions were subjected to repeated chromatography using silica gel, Sephadex^®^ LH-20, PTLC to afford ten compounds: **6** (14 mg), **7** (9 mg), **8** (60 mg), **9** (51 mg), **10** (35 mg), **11** (16 mg), **12** (29 mg), **13** (61 mg), **14** (10 mg) and **15** (10 mg). ^1^H- and ^13^C-NMR and 2D spectra of the isolated compounds were recorded using a Bruker DRX 600 NMR spectrometer (Bruker Daltonics Inc., Billerica, MA, USA).

### 3.3. Acetylcholine Esterase Inhibitory Assay

AChEI activity was measured using Ellman’s method [35,36]. AChE hydrolyzes the substrate ACTI into acetate and thiocholine. In a neutral and alkaline medium; thiocholine reacts with DTNB to give yellow colored 2-nitro-5-thiobenzoate, which can be detected spectrophotometrically at 405 nm. Briefly, in a 96-well plate, 25 µL of 15 mM ACTI, 125 µL of 3 mM DTNB in buffer B (50 mM Tris HCl, pH = 8, 0.1 M NaCl, 0.02 M MgCl_2_·6H_2_O), 50 µL of buffer A (50 mM Tris-HCl, PH 8, 0.1% BSA) and 25 µL of tested sample (dissolved in 25% DMSO) were mixed, and the absorbance was measured using a microplate reader (Biotek, Winooski, VT, USA) at 405 nm every 16 s ten times. Then, 25 µL of AChE (0.25 U/mL in buffer A) was added and the absorbance was measured ten times every 16 s. A solution of 25% DMSO was used as a negative control. Absorbance was plotted against time and enzyme activity was calculated from the slope of the line so obtained and expressed as a percentage compared to an assay using a buffer without any inhibitor. Fractions were tested at 400 µg/mL. To avoid any increase in absorbance due to the color of the compounds or spontaneous hydrolysis of the substrate, the absorbance before addition of the enzyme was subtracted from the absorbance after adding the enzyme. 

### 3.4. Cell Line Assays

Neuro-2A cell line was maintained in Eagle’s Minimum Essential medium supplemented with 10% fetal bovine serum, 1% of 100× antibiotic—antimitotic agents (containing penicillin and streptomycin) in a humid atmosphere of 5% (v/v) CO_2_ and 95% (v/v) air at 37 °C. Neuro-2A cells were plated in 96-well cell culture plates at a density of 3 × 10^4^ cells/well for H_2_O_2_ and intracellular ROS assays and 2.5 × 10^3^ cells/well for the Aβ_25-35_-assay.

#### 3.4.1. Determination of Cell Viability 

Cell viability was determined by MTT reduction assay. Briefly, after treatment with isolated compounds, the media was replaced by fresh one after washing the cells with 100 µL PBS and 20 µL of MTT (5 mg/mL) was added to each well. After 4 h incubation at 37 °C, the state of cells was checked under microscope to confirm either cytotoxicity in H_2_O_2_ and Aβ_25-35_ wells or protection in positive controls and isolated compounds ones. The supernatants were aspirated carefully, and 150 µL of DMSO was added to each well to dissolve the dark blue formazan crystals. The absorbance was measured using a microplate reader at 570 nm. 

#### 3.4.2. Protection against H_2_O_2_-Induced Neurotoxicity 

H_2_O_2_ interacts with metals in the medium, producing highly cytotoxic hydroxyl radicals that induce neurotoxicity. So, active compounds are those that can scavenge free radicals and increase the cell viability. This assay was performed as previously described [37] with slight modifications. Briefly, the concentration of H_2_O_2_ that decreased cell viability to about 50% was determined as follows: cells were cultured in a 96-well plate and after 24 h were further incubated with different concentrations of H_2_O_2_ (50, 100, 150, 200 µM) freshly prepared from 30% stock solution. After 24 h, cell viability was determined using the MTT reduction method. H_2_O_2_ at a concentration of 150 µM had the desired cytotoxicity and was used for the next step. Cells were incubated with different concentrations (6.25, 12.5, 25, 50 and 100 µM) of the isolated compounds and catechin [38] as a positive control (fractions were tested at 400 µg/mL, the maximum solubility) for 2 h followed by H_2_O_2_ treatment for 24 h.

#### 3.4.3. Protection against Aβ_25-35_-Induced Neurotoxicity

Similarly, the concentration of Aβ_25-35_ that decreased cell viability to about 50% was determined. It was found that 25 µM of Aβ_25-35_ had the desired cytotoxicity after 72-h incubation. So, the cells were incubated with different concentrations of the isolated compounds (6.25, 12.5, 25, 50 and 100 µM) and EGCG as a positive control [39] for 3 h followed by Aβ_25-35_ treatment for 72 h. 

#### 3.4.4. Scavenging of Intracellular Reactive Oxygen Species (ROS)

This assay depends on using Bes-H_2_O_2_-AC, a highly selective probe for H_2_O_2_ that can permeate through the cell membrane and deacetylated inside the cells to give an impermeable product, Bes-H_2_O_2_; this is then oxidized by intracellular ROS to give a fluorescent product; difluorofluorescein (DFF). Increased fluorescence means increased oxidative stress, and active compounds are those that can scavenge ROS and decrease the fluorescence intensity. This assay was performed as previously described [40] with some modifications. Briefly, Neuro-2A cells were seeded in 96-well Clear Fluorescence Black Plate (number 655090, Greiner Bio-one, Tokyo, Japan). Cells were treated with three different concentrations (25, 50, 100 µM) of the samples. After 2 h incubation, cells were subjected to oxidative stress using 600 µM of H_2_O_2_ for 30 min. The nucleus was stained by Hoechst 33342 (Dojindo, Kumamoto, Japan), and the amount of difluorofluorescein (DFF) released from the reaction of H_2_O_2_ and BES-H_2_O_2_-Ac was taken as an indication of the amount of intracellular H_2_O_2_ and ROS. Images of each well were acquired using an IN Cell Analyzer 1000 (GE Healthcare, Buckinghamshire, UK) using 360-nm (Hoechst 33342) and 480-nm (BES-H_2_O_2_-Ac) excitation filters and monitored through 460-nm and 535-nm emission filters, respectively. Developer software was used to analyze the images of Hoechst 33342 and BES-H_2_O_2_-Ac staining, and the resulting data were then applied to Spotfire Decision Site Client 8.2 software for visualizing the results. 

### 3.5. Scavenging of Hydroxyl Radical Using 2-Deoxyribose Degradation Assay

The inhibitory effect of bioactive compounds on deoxyribose degradation was determined by measuring the competition between deoxyribose and bioactive compounds for the hydroxyl radicals (^.^OH) generated from the Fe^3+^/ascorbate/EDTA/H_2_O_2_ system (referred to non-site-specific assay; the major activity measured is ^.^OH scavenging) or an Fe^3+^/ascorbate/H_2_O_2_ system (referred to as a site-specific assay; major activity measured is metal chelation) [41,42]. Both assays depend on the formation of a highly reactive hydroxyl radical, OH, by the interaction of iron ions with H_2_O_2_ through Fenton’s reaction. Then the pentose sugar; 2-deoxyribose is attacked by^.^OH radicals to yield malondialdehyde bis-dimethyl acetal which is thiobarbituric acid reactive substance (TBARS). On heating with thiobarbituric acid form a pink chromogen that can be measured by its absorbance at 532 nm. This assay was performed as previously described [43]. Different concentrations of tested compounds were dissolved in 400 µL phosphate buffer (0.2 M, pH 7.4). Then, 50 µL of deoxyribose (60 mM), 50 µL of Na_2_EDTA (2 mM), 50 µL of FeCl_3_ (3.2 mM), 50 µL of H_2_O_2_ (60 mM) and 50 µL of ascorbic acid (2 mM) were added, and the total volume of the reaction mixture was adjusted to 1 mL with buffer. After water-bath incubation at 50 °C for 25 min, the reaction was terminated by 250 µL of trichloroacetic acid (10%, w/w). The pink color was obtained after addition of 150 µL of TBA (5%, in 1.25% NaOH aqueous solution) and heating in an oven at 105 °C for 25 min. After cooling, the absorbance was measured at 532 nm against a blank containing all the reaction mixtures except the sample. For site-specific hydroxyl-radical-scavenging activity, the reaction mixture was similar to the above method, except that EDTA was replaced by an equivalent volume of buffer. Percent inhibition of deoxyribose degradation was calculated with the equation % inhibition = (1 − Abs. of tested sample/Abs. of control) × 100.

### 3.6. Thioflavin (ThT) Assay 

Thioflavin T (ThT), abenzothiazole salt, is used to quantify misfolded protein aggregates and amyloid peptides [44]. ThT is a non-fluorescent dye which, when it binds to amyloid rich aggregates, produces a fluorescent signal proportional to the quantity of amyloid aggregation [45]. Aβ_25-35_ (25 µM) freshly prepared in PBS (50 mM, pH = 7.4) was incubated at 37 °C with or without different concentrations (100, 50, 25 µM) of isolated compounds at 37 °C and shaken at 300 rpm for 3 h. One hundred µL of each incubated sample was transferred to a 96-well plate, and five µM ThT was added. The ThT fluorescence was measured over 180 min at 10-min intervals at emission λ at 485 nm; and excitation λ at 442 nm using FlexStation 3 Microplate Reader (Molecular Devices, LLC, Sunnyvale, CA, USA), and data were managed by SoftMax Pro^®^ 5.4.1 software. The samples were constantly shaken every 60 sec during the incubation. 1% DMSO and different concentrations of ECCG were taken as negative and positive controls, respectively. The area under the curve (AUC) was taken as indicative of fluorescence intensity. Fluorescence of samples and ThT without Aβ_25-35_ (sample blank) was detected and subtracted from the final AUC to prevent any interference. 

### 3.7. Molecular Docking 

Docking experiment was used to investigate the binding affinity of the active compounds to the binding residues of the active site of AChE. The crystal structure of aged phosphonylated AChE was downloaded from the RCSB Protein Data Bank (PDB, code 1CFJ) and imported into the work place of Molegro Virtual Docker (MVD 6.0, Molegro, Aarhus, Denmark) software. All cofactors, ligands and water molecules were removed from the protein structure before docking. The docking experiment was adjusted as previously described [46]. The three-dimensional structures of ligands were drawn using Chemsketch 12.0 software (Cambridge Soft Corporation, Cambridge, MA, USA) and saved as mol2 formats. compounds were docked inside a sphere with a 15 °A radius centered at the largest cavity (volume 228.352, surface 546.56 °A) detected by the program. Steps of docking experiment were done as previously described [47]. The binding residues were determined using LigPlot^+^ V.1.4 program.

### 3.8. Statistical Analysis

IC_50_ values of isolated compounds in AChEI and scavenging of hydroxyl radical assays were calculated using Probit Analysis (SPSS Version 16.0 for Windows, SPSS Inc., Chicago, IL, USA) for five different concentrations in three independent experiments. Cell viability was expressed as a percentage of MTT reduction, assuming that the viability of cells treated with 1% DMSO as a negative control was 100%. Figures were built in Microsoft Excel 2010. All values are the mean ± SD of three independent experiments. Data were analyzed for statistical significance using one-way ANOVA, followed by Dunnet’s test as a post-hoc test with GraphPad Prism 5.0 software for Windows (Inc., San Diego, CA, USA).

**Table 4:** Summary of the activity of the isolated compounds in the tested experiments.

Compound	AchEI IC_50_(µM)	Protection against H_2_O_2_	Hydroxyl Radical Scavenging/IC_50_ (µM)	Intracellular ROS Scavenging	Protection against Aβ_25-35_	Decrease of ThT Fluorescence
α-Glutinol (**1**)	-	-	-	-	-	-
β-amyrin (**2**)	-	-	-	-	-	-
Isoepoxypteryxin (**3**)	327.4 ± 3.4	-	-	-	++	++
Isopteryxin (**4**)	475.9 ± 1.5	-	-	-	+	+
β--Sitosterol-3-*O*-glucoside (**5**)	-	-	-	-	-	-
Hyuganin E (**6**)	286.5 ± 2.1	+	+	+	++	++
5-(Hydroxy methyl)-2-furaldehyde (**7**)	-	-	-	-	-	-
Kaempferol (**8**)	>500	+	720 ± 7.9	+	+	+
Luteolin (**9**)	>500	++	753 ± 11.1	++	+	-
Methyl chlorogenate (**10**)	>500	++	840 ± 12.4	++	-	-
Chlorogenic acid (**11**)	-	++	602 ± 8.9	++	-	-
Quercetin (**12**)	35.5 ± 1.3	++	672 ± 10.6	++	+	-
Kaempferol-3-*O*-glucoside (**13**)	80.4 ± 5.1	+	1150 ± 6.7	+	+	+
Kaempferol-3-*O*-rutinoside (**14**)	50.4 ± 0.4	+	1030 ± 25.7	+	++	++
Adenosine (**15**)	-	-	-	-	-	-

+ Weak activity, ++ Strong activity.

## 4. Conclusions 

In conclusion, this study characterized the neuroprotective compounds of *A. shikokiana* (Table 4). The isolated compounds quercetin, luteolin, chlorogenic acid and its methyl ester could protect against H_2_O_2-_induced neurotoxicity by decreasing oxidative stress through scavenging of hydroxyl radicals and intracellular ROS. Quercetin, kaempferol glycosides and isolated coumarins could inhibit AChE through binding to the active site of AChE. Kaempferol rutinoside, hyuganin E and isoepoxypteryxin could protect against Aβ*-*induced neurotoxicity. Further investigation of the *in vivo* neuroprotective activity of the aerial parts is needed to provide a complete profile of its activity. As there are no previous reports about neuroprotection of isoepoxypteryxin and hyuganin E, we recommend the investigation of the *in vivo* activity, pharmacokinetic and pharmacodynamics studies of those compounds. 
